# “Adopt-a-Tissue” Initiative Advances Efforts to Identify Tissue-Specific Histone Marks in the Mare

**DOI:** 10.3389/fgene.2021.649959

**Published:** 2021-03-26

**Authors:** N. B. Kingsley, Natasha A. Hamilton, Gabriella Lindgren, Ludovic Orlando, Ernie Bailey, Samantha Brooks, Molly McCue, T. S. Kalbfleisch, James N. MacLeod, Jessica L. Petersen, Carrie J. Finno, Rebecca R. Bellone

**Affiliations:** ^1^Veterinary Genetics Laboratory, School of Veterinary Medicine, University of California, Davis, Davis, CA, United States; ^2^Department of Population Health and Reproduction, School of Veterinary Medicine, University of California, Davis, Davis, CA, United States; ^3^Faculty of Science, School of Life and Environmental Science, University of Sydney, Camperdown, NSW, Australia; ^4^Department of Animal Breeding and Genetics, Swedish University of Agricultural Sciences, Uppsala, Sweden; ^5^Livestock Genetics, Department of Biosystems, KU Leuven, Leuven, Belgium; ^6^Centre d'Anthropobiologie et Génomique de Toulouse (CAGT), Faculté de Médecine Purpan, Université Toulouse III-Paul Sabatier, Toulouse, France; ^7^Maxwell H. Gluck Equine Research Center, Department of Veterinary Science, University of Kentucky, Lexington, KY, United States; ^8^Department of Animal Sciences, University of Florida, Gainesville, FL, United States; ^9^Department of Veterinary Population Medicine, College of Veterinary Medicine, University of Minnesota, St. Paul, MN, United States; ^10^Department of Animal Science, University of Nebraska-Lincoln, Lincoln, NE, United States

**Keywords:** genome, annotation, epigenetics, horse, chromatin, consortium, collaboration, regulation

## Introduction

The equine genetics and genomics research community has a long history of synergistic collaborations for developing tools and resources to advance equine biology. Starting in 1995 with the first International Equine Gene Mapping Workshop supported by the Dorothy Russell Havemeyer Foundation Inc. (Bailey, 2010), researchers collaborated to build comprehensive equine linkage maps (Guérin et al., 1999, 2003; Penedo et al., 2005; Swinburne et al., 2006), radiation hybrid and comparative maps (Caetano et al., 1999; Chowdhary et al., 2002), physical marker and BAC contig maps (Raudsepp et al., 2004, 2008; Leeb et al., 2006), reference genomes for the horse (Wade et al., 2009; Kalbfleisch et al., 2018), and genotyping arrays to economically map and study traits of interest for horse owners and breeders (McCue et al., 2012; McCoy and McCue, 2014; Schaefer et al., 2017). Continuing the legacy of community-based advancements, a new collective effort began in 2015 to functionally annotate DNA elements in the horse as part of the international Functional Annotation of ANimal Genomes (FAANG) Consortium (Andersson et al., 2015; Tuggle et al., 2016; Burns et al., 2018).

Reminiscent of the ENCODE project in humans and mice (Dunham et al., 2012), the ultimate goal of the FAANG consortium is to annotate the major functional elements in the genomes of domesticated animal species (Andersson et al., 2015). In particular, four histone modifications were chosen by the consortium to characterize the genomic locations of enhancers (H3K4me1), promoters and transcription start sites (H3K4me3), open chromatin with active regulatory elements (H3K27ac), and facultative heterochromatin with inaccessible or repressed regulatory elements (H3K27me3) (Andersson et al., 2015; Giuffra and Tuggle, 2019). The initial equine FAANG efforts identified putative regulatory regions in eight prioritized tissues of interest (TOI) by performing Chromatin Immuno-Precipitation Sequencing (ChIP-Seq) for the four target histone marks (Kingsley et al., 2020). In that investigation, more than one million putative regulatory sites were characterized across the equine genome. With more than 80 tissues, cell lines, and body fluids stored in the equine biobank (Burns et al., 2018), further opportunities to expand the scope of the annotation work exist. To leverage the benefits of the biobank, a collaborative sponsorship program titled “Adopt-a-Tissue” was created to enable researchers from across the globe to select and support annotation of a tissue by the equine FAANG group. Through this effort, four additional “Adopted” tissues— spleen, metacarpal 3 (MC3), sesamoid, and full thickness skin— were assayed by histone mark ChIP-Seq to expand the tissue-specific annotation resources for the entire equine research community.

## Methods

All ChIP-Seq assays were performed by Diagenode ChIP-Seq Profiling Service (Diagenode, Cat# G02010000, Liège, Belgium). Summarized experimental procedures are available in more detail at the FAANG FTP portal hosted by EBI (ftp://ftp.faang.ebi.ac.uk/faang/ftp/protocols/assays/ and ftp://ftp.faang.ebi.ac.uk/faang/ftp/protocols/experiments/). Spleen samples were processed following the assay procedures outlined in UCD_SOP_ChIP-Seq_for_Histone_Marks_20191101.pdf. Skin and both bone tissues were processed following the experimental protocols outlined in UCD_SOP_ChIP-seq_for_Histone_Marks_Skin_20201218.pdf and UCD_SOP_ChIP-seq_for_Histone_Marks_Bone_20201218.pdf, respectively. “Adopted” tissues, as summarized in Supplementary Table 1, were collected from two Thoroughbred mares (denoted as ECA_UCD_AH1 for SAMEA104728862 and ECA_UCD_AH2 for SAMEA104728877) as part of the FAANG equine biobank (Burns et al., 2018) following protocols approved by the University of California, Davis Institutional Animal Care and Use Committee (Protocol #19037).

Chromatin was isolated from the two bone tissues using the TrueMicro ChIP-Seq kit (Diagenode Cat# C01010140) and from spleen and skin using the iDeal ChIP-Seq kit for Histones (Diagenode Cat# C01010059). Starting amounts for each replicate varied by tissue with ~100 mg for spleen, 375–770 mg for MC3, 445–650 mg for sesamoid, and ~125 mg for skin. After homogenization, fixed samples were sheared with the Bioruptor® Pico (Diagenode Cat# B01060001) for 12 (spleen), 10–12 (MC3 and sesamoid), and 8 (skin) cycles of 30 s on and 30 s off. The amount of chromatin yield and thus chromatin per IP varied by tissue. Spleen and skin had the greatest amounts (1.5 μg and 600 ng, respectively) per IP and MC3 and sesamoid had the least (350 ng each). The following antibody concentrations were used for MC3, sesamoid, and skin: 0.5 μg for H3K4me1, 0.5 μg for H3K4me3, 1 μg for H3K27ac, and 1 μg for H3K27me3. To account for the greater amount of chromatin from spleen, twice the amount of antibody was used for each mark compared to the other three tissues. For all tissues, 10% of the total chromatin from each replicate was saved for the input.

Libraries were prepared with the IP-Star® Compact Automated System (Diagenode Cat# B03000002) using the MicroPlex Library Preparation Kit v2 (Diagenode Cat# C05010013). Spleen, MC3, and sesamoid were sequenced as 50 base pair single-end (SE) reads on the HiSeq 4000 platform (Illumina, San Diego, CA, USA). For these tissues, the broad mark (H3K27me3) was sequenced to a minimum of 50M raw reads while the remaining marks (H3Kme1, H3K4me3, and H3K27ac) and the input were sequenced to a minimum depth of 30 M raw reads. Due to advancements in sequencing technology, skin tissue was sequenced as 50 base pair paired-end (PE) reads on the NovaSeq 6000 (Illumina, San Diego, CA, USA). For skin, the broad mark (H3K27me3) was sequenced to a minimum of 100 M raw fragments while the remaining marks (H3Kme1, H3K4me3, and H3K27ac) and the input were sequenced to a minimum depth of 40 M raw fragments.

Methods for analyzing SE reads followed the procedures described previously (Kingsley et al., 2020) and modifications were made to the SE analysis methods to accommodate PE data generated from skin. After trimming with Trim-Galore version 0.4.0 (Martin, 2011; Andrews et al., 2012), reads were aligned to EquCab3.0 (Kalbfleisch et al., 2018) with BWA-MEM version 0.7.9a (Li and Durbin, 2009). Alignments in BAM format were filtered using SAMtools version 1.9 (Li et al., 2009). Reads were removed if they did not map, had secondary alignments (including split hits), failed platform/vendor quality tests, were identified as optical duplicates, or had an alignment quality score <30. PE reads were also removed if the mates did not map. PCR duplicates were marked with PicardTools version 2.7.1 (Picard toolkit, 2019) and removed with SAMtools. For peak-calling, MACS2 version 2.1.1.20160309 (Zhang et al., 2008) was used to call peaks for all marks with PE data denoted by a PE flag (-f BAMPE). SICERpy version 0.1.1 was also used to call peaks for H3K27me3 as it specializes in broad peak calling (SICERpy, SICERpy, GitHub Repository; Zang et al., 2009). To use SICERpy with the PE data, the second read in each pair was removed and data were processed as SE based on recommendations from the software developers. Peak-calls were combined by identifying overlapping regions of enrichment in both biological replicates where at least one replicate was significantly enriched for a given mark. Heatmaps and quality metrics were generated using deepTools 2.4.2 (Ramírez et al., 2016), SPP 1.13 (Kharchenko et al., 2008), and custom scripts. Detailed bioinformatic workflows are available at ftp://ftp.faang.ebi.ac.uk/faang/ftp/protocols/analysis/.

## Quality Assessment

### Library Complexity

Data were assessed for library complexity with metrics established by ENCODE and endorsed by FAANG, including nonredundant fraction (NRF), PCR bottleneck coefficient 1 (PBC1), and PCR bottleneck coefficient 2 (PBC2) (Landt et al., 2012; Kingsley et al., 2020). All of the libraries prepared surpassed the quality threshold for the PBC2 metric (PBC2 > 1), however, several marks and tissues fell below the quality threshold for NRF and PBC1 (Table 1). For example, three of the four marks for spleen passed all library complexity measures while the H3K27me3 data from both biological replicates failed NRF and PBC1. Additionally, both replicates for sesamoid and MC3 passed all three metrics for H3K4me1 and H3K27me3 but fell below threshold for H3K4me3 and H3K27ac. All skin libraries passed NRF and PBC1 thresholds with three exceptions: both replicates for H3K4me3 and ECA_UCD_AH2 replicate for H3K4me1.

**Table 1:** Quality metrics and peak-calling summary for each biological replicate.

**Mark**	**Tissue**	**Replicate**	**NRF**	**PBC1**	**PBC2**	**NSC**	**RSC**	**JSD**	**FRiP**	**Peak calls**
		**Threshold:**	**(>0.5)**	**(>0.5)**	**(>1)**	**(>1.05)**	**(>0.8)**	**(>0.05)**	**(>0.01)**	
K4me1	Spleen	AH1	0.65	0.64	2.82	1.03	0.98	0.39	0.20	84,146
K4me1	Spleen	AH2	0.74	0.74	3.80	1.04	0.96	0.39	0.25	113,447
K4me3	Spleen	AH1	0.62	0.64	2.98	2.67	1.44	0.62	0.57	28,735
K4me3	Spleen	AH2	0.57	0.60	2.67	2.53	1.34	0.62	0.57	31,198
K27ac	Spleen	AH1	0.65	0.66	2.96	1.34	1.54	0.46	0.29	50,977
K27ac	Spleen	AH2	0.70	0.70	3.40	1.31	1.46	0.45	0.29	60,281
K27me3	OriginalSpleen	AH1	0.43	0.42	1.79	1.01	0.64	0.02	0.05	1,136
K27me3	RepeatSpleen	AH1	0.13	0.28	2.94	1.03	0.33	0.20	0.02	164
K27me3	MergedSpleen	AH1	0.32	0.40	1.86	1.01	0.59	0.50	0.05	6,297
K27me3	OriginalSpleen	AH2	0.44	0.43	1.82	1.01	0.67	0.05	0.08	6,647
K27me3	RepeatSpleen	AH2	0.66	0.67	3.02	1.02	0.74	0.06	0.10	32,492
K27me3	MergedSpleen	AH2	0.53	0.55	2.40	1.01	0.83	0.06	0.11	37,629
K4me1	Sesamoid	AH1	0.63	0.63	2.64	1.02	0.63	0.31	0.08	43,397
K4me1	Sesamoid	AH2	0.84	0.85	6.46	1.01	0.49	0.21	0.00	4
K4me3	Sesamoid	AH1	0.37	0.39	1.79	2.33	1.26	0.57	0.48	19,617
K4me3	Sesamoid	AH2	0.39	0.40	1.78	1.40	1.20	0.41	0.21	16,524
K27ac	Sesamoid	AH1	0.42	0.42	1.81	1.16	1.19	0.40	0.19	34,223
K27ac	Sesamoid	AH2	0.34	0.34	1.67	1.02	0.60	0.31	0.02	5,013
K27me3	Sesamoid	AH1	0.68	0.68	3.11	1.01	0.48	0.25	0.06	1,840
K27me3	Sesamoid	AH2	0.75	0.75	3.97	1.01	0.56	0.18	0.00	0
K4me1	MC3	AH1	0.74	0.74	3.76	1.02	0.68	0.12	0.09	56,238
K4me1	MC3	AH2	0.77	0.77	4.28	1.02	0.63	0.13	0.07	47,452
K4me3	MC3	AH1	0.14	0.27	2.91	2.73	1.22	0.48	0.50	19,209
K4me3	MC3	AH2	0.32	0.34	1.71	2.56	1.20	0.48	0.50	21,339
K27ac	MC3	AH1	0.27	0.29	1.65	1.25	1.19	0.30	0.23	36,022
K27ac	MC3	AH2	0.09	0.26	4.56	1.38	0.95	0.32	0.19	16,638
K27me3	MC3	AH1	0.57	0.58	2.36	1.01	0.55	0.25	0.10	17,001
K27me3	MC3	AH2	0.65	0.65	2.88	1.01	0.51	0.22	0.08	13,790
K4me1	Skin	AH1	0.43	0.47	2.14	1.15	2.85	0.29	0.34	115,470
K4me1	Skin	AH2	0.53	0.55	2.39	1.13	3.06	0.25	0.29	109,322
K4me3	Skin	AH1	0.41	0.46	2.12	3.14	1.27	0.60	0.69	24,442
K4me3	Skin	AH2	0.32	0.40	2.12	3.19	1.30	0.60	0.68	23,584
K27ac	Skin	AH1	0.50	0.53	2.30	1.51	1.45	0.41	0.47	58,278
K27ac	Skin	AH2	0.58	0.59	2.57	1.47	1.46	0.40	0.47	57,737
K27me3	Skin	AH1	0.50	0.53	2.36	1.06	3.20	0.14	0.24	95,788
K27me3	Skin	AH2	0.50	0.54	2.40	1.06	4.09	0.11	0.21	77,151

*The summary includes six quality metrics—NRF, Non-Redundant Fraction; PBC1 and PBC2, PCR Bottleneck Coefficient 1 and 2; NSC, Normalized Strand Cross-Correlation Coefficient; RSC, Relative Strand Cross-Correlation Coefficient; FRiP, Fraction of Reads in Peaks– and thresholds originally established by ENCODE, and the Jensen Shannon Distance (JSD). Samples include all of the original spleen IP, the repeated spleen IP for H3K27me3, and the merged (original + repeated) spleen IPs for H3K27me3. Peaks used to determine FRiP and peak numbers for H3K27me3 were called with SICER. All other peaks were generated with MACS2. Biological replicates are denoted as AH1 for SAMEA104728862 and AH2 for SAMEA104728877. Red highlighting indicates values below the quality thresholds*.

In addition to quality metrics, sequencing data were evaluated at several processing stages of the analysis including alignment and PCR deduplication. All datasets generated high mapping quality scores (>35) and exceeded the minimum sequencing targets as described in the methods (Supplementary Table 2). Skin and spleen tissues retained a high number of reads for H3K4me1, H3K4me3, and H3K27ac after alignment, filtering, and deduplication (>20 M reads per replicate). Although all three activating marks were sequenced to the same target for both bone tissues, H3K4me1 retained more than 20 M reads per replicate while H3K4me3 and H3K27ac fell below 20 M processed reads per replicate with the majority of reads removed by deduplication. More than 40 M reads remained for each H3K27me3 replicate after processing with the exception of ECA_UCD_AH2 for sesamoid.

### IP Enrichment

Data were also evaluated for IP enrichment using a variety of metrics to determine signal quality. Using normalized strand cross correlation (NSC) and relative strand cross correlation (RSC) assessments established by ENCODE (Landt et al., 2012), all marks for skin tissue exceeded the minimum quality threshold (Table 1). Additionally, the biological replicates for H3K4me3 and H3K27ac from spleen and MC3, as well as the H3K4me3 replicates for sesamoid, passed both cross-correlation measures. Similar to the library complexity metrics, several tissues fell below the quality thresholds (NCS > 1.05 and RSC > 0.8) including H3K4me1 from sesamoid and MC3; H3K27ac from ECA_UCD_AH2 sesamoid; and H3K27me3 from spleen, sesamoid, and MC3. Alignments were also assessed using the Jensen Shannon distance (JSD) to compare the distribution of reads with that of the background (input). Using JSD, H3K27me3 from both spleen replicates had values below 0.05, which is indicative of insufficient IP enrichment.

The final measure of IP enrichment evaluated the fraction of reads in peaks (FRiP) by comparing the peak calls with the read distribution for each sample. All tissues produced a high proportion of aligned reads within peaks for H3K4me3, ranging from 0.21 for sesamoid to 0.69 for skin. Similarly, MC3, skin, and spleen generated high FRiP scores for H3K27ac (0.47–0.19), and peaks from skin and spleen also scored well for H3K4me1 (0.47–0.29). Although lower than the values from skin and spleen, FRiP scores from MC3 indicated sufficient enrichment was obtained for H3K4me1 (0.07–0.09). For sesamoid tissue, the ECA_UCD_AH2 replicate generated peaks with comparable enrichment for H3K4me1, H3K27ac, and H3K27me3, while the ECA_UCD_AH2 replicate scored below threshold for both H3K4me1 and H3K27me3 (0.0005 and 0.0043, respectively). Further, H3K27me3 peaks from skin generated a substantially higher fraction of reads compared with MC3 and spleen (0.21–0.24 vs. 0.05–0.10), although all three of these tissues obtained sufficient enrichment based on this assessment.

### Replicate Comparison

In addition to quality assessments for the read alignments, peaks called from the biological replicates were compared. For most of the marks, the percentage of genome covered by peaks was consistent with previously reported values for the TOI (Table 1). For sesamoid tissue, at least one replicate for H3K4me1, H3K27ac, and H3K27me3 generated fewer peak calls than expected based on results from the other replicate and the MC3 replicates. Additionally, the initial data for H3K27me3 from both spleen replicates yielded fewer peaks in accordance with the low complexity and enrichment scores for those libraries. The Jaccard similarity coefficient identified the highest correlation between the biological replicates for H3K4me3 across all “adopted” tissues, ranging from 0.65 to 0.84 (Table 2), and data from skin also showed high correlation for all marks (0.44–0.84). Replicates for spleen and MC3 had moderate levels of similarity for H3K4me1 and H3K27ac (0.32–0.58), while the biological replicates for H3K4me1 and H3K27me3 from sesamoid had no identity detected, consistent with the low-scoring quality assessments.

**Table 2:** Summary of the combined peak calls and replicate comparison.

**Mark**	**Tissue**	**Combined peak number**	**% Covered**	**Jaccard similarity coefficient**
H3K4me1	Spleen	73,528	2.98	0.44
H3K4me3	Spleen	28,661	1.56	0.80
H3K27ac	Spleen	51,427	1.82	0.58
H3K27me3 MACS2	Spleen	7,349	0.09	0.01
H3K27me3 SICER	Spleen	449	0.22	0.03
H3K4me1	MC3	46,511	1.16	0.32
H3K4me3	MC3	20,556	1.10	0.75
H3K27ac	MC3	31,547	1.08	0.38
H3K27me3 MACS2	MC3	15,304	0.40	0.28
H3K27me3 SICER	MC3	5,628	2.57	0.28
H3K4me1	Sesamoid	750	0.01	0.00
H3K4me3	Sesamoid	17,361	1.07	0.65
H3K27ac	Sesamoid	13,160	0.67	0.08
H3K27me3 MACS2	Sesamoid	390	0.01	0.00
H3K27me3 SICER	Sesamoid	703	0.26	0.00
H3K4me1	Skin	92,971	4.56	0.50
H3K4me3	Skin	24,353	1.60	0.84
H3K27ac	Skin	54,946	3.38	0.67
H3K27me3 MACS2	Skin	51,480	6.02	0.44
H3K27me3 SICER	Skin	11,764	6.28	0.44

*The summary includes the combined number of peaks and the percentage of the genome covered by those peaks. The Jaccard Similarity Coefficient compares the two biological replicates with 1 being perfectly concordant and 0 being entirely discordant. Peaks for H3K4me1, H3K4me3, and H3K27ac were called with MACS2*.

### Additional Data Collection

Due to insufficient enrichment and replicate identity, IP and sequencing were repeated for H3K27me3 from both spleen replicates. Unfortunately, the repeated ECA_UCD_AH1 data had low library complexity and IP enrichment (Table 1 and Supplementary Table 2). To achieve sufficient data for accurate peak calling from spleen tissue, the first round of IP and sequencing from ECA_UCD_AH1 for H3K27me3 and both rounds from ECA_UCD_AH2 were used for combined peak calling. Reads from the two input files for ECA_UCD_AH2 were also merged. The number of combined peaks increased from 4,955 covering 1.98% from the first round of sequencing to 5,267 covering 2.18% of the genome when data were merged (Table 2). Similar issues with enrichment prevented sufficient signal for peak calling in sesamoid for three of the four marks, and therefore, a second round of IP and quality evaluation of ECA_UCD_AH2 sesamoid is underway for H3K4me1, H3K27ac, and H3K27me3.

## Data Metrics

After combining replicates, the number of retained peaks for each mark from the SE data ranged from 4,933 to 73,528 for spleen and from 5,628 to 46,511 for MC3 (Table 2). For both tissues, H3K4me1— the mark indicative of enhancers— was found to have the highest number of peaks while the repressive mark was found to have the lowest. This pattern is also consistent with the TOI data (Kingsley et al., 2020). For PE skin data, the number of combined peaks varied from 24,353 to 92,971 regions, and H3K4me3, which denotes promoters, was the mark with the lowest number of peaks. Additionally, the amount of the genome covered by H3K27me3 peaks was substantially higher for skin compared to the other equine FAANG tissues analyzed to date (6.28 vs. 2.94%), while the number of reads retained for H3K27me3 from the PE data after filtering (42.8%) was comparable to the average retained for all of the equine H3K27me3 SE data (41.3%, PRJEB42315 and PRJEB35307).

Evaluating general enrichment patterns revealed that the “adopted” tissues detected mark distributions for the activating marks that were consistent with those identified previously for the TOI (Supplementary Figures 1–3). Data for H3K27me3 from skin, however, generated strong enrichment around the TSS and upstream of an average gene, while still maintaining a similar level of relative enrichment for H3K27me3 distributed throughout the rest of the gene body and downstream as seen for other tissues (Supplementary Figure 4). Evaluation of the spleen datasets detected the strongest H3K27me3 enrichment when combining the original ECA_UCD_AH1 dataset and the merged ECA_UCD_AH2 dataset (denoted as “spleen” on Supplementary Figure 4). While enrichment distributions for sesamoid tissue detected consistent patterns for H3K4me1, H3K27ac, and H3K27me3, the relative level of enrichment is lower than expected based on the other tissues. In addition to genome-wide evaluations, the replicate-combined peak calls were also manually evaluated across a small number of well-characterized regions. Consistent with expectations, activating marks were detected at the TSS and upstream of ubiquitously expressed genes such as *ACTB* for all tissues (Supplementary Figures 5A,B). Additionally, all “adopted” tissues lacked peaks indicative of active transcription for a liver-specific gene known as *CYP2E1* (Supplementary Figures 5C,D).

## Discussion

The ENCODE project profoundly impacted scientific understanding of genome function in humans by enabling researchers to explore previously impossible challenges, such as charting genomic landscape shifts during development and uncovering enhancer networks associated with disease (Nord et al., 2013; Rhie et al., 2016). The advancements made by ENCODE paved a path for the FAANG consortium to characterize genomic function in numerous agricultural species (Andersson et al., 2015; Tuggle et al., 2016; Giuffra and Tuggle, 2019), which will expand research opportunities across diverse genera. As a part of the larger consortium, the equine FAANG group established a community-based initiative to “adopt” additional tissues for annotation. As a result of that expansive collaborative effort, characterization of putative regulatory regions was performed in spleen, sesamoid, MC3, and skin. The four additional tissues are of major importance for equine health and traits of economic impact. Specifically, research on catastrophic fracture involving sesamoid and MC3 can benefit from bone-specific annotations as recent advances in treatment have focused on transgenically modified stem cell therapeutics (Ball et al., 2019). Similarly, many diseases and traits under artificial selection in horses, such as melanoma, insect bite hypersensitivity, and coat colors including Appaloosa spotting among others, involve skin tissue (Rieder et al., 2000, 2001; Bellone et al., 2008, 2013; Rosengren Pielberg et al., 2008; Curik et al., 2013; Lanz et al., 2017). Several of these characterized phenotypes have been associated with mutations affecting gene expression (Rieder et al., 2000; Rosengren Pielberg et al., 2008; Bellone et al., 2013), making regulatory regions identified from whole skin a valuable resource for equine researchers. The “Adopt-a-Tissue” effort fits into a broader legacy of collaborative resource development that has historically led to rapid advancements for equine genomics and will continue to push equine science toward new frontiers. In concordance with past community efforts, the high quality data generated from the “Adopted” tissues are publicly available to benefit all investigators and lead to further progress in equine research.

Using quality metrics first standardized by ENCODE (Dunham et al., 2012), we identified low IP enrichment for the broad mark in spleen, sesamoid, and MC3 tissues. Unlike the SE datasets, the skin replicates sequenced with PE reads generated a higher enrichment signal for H3K27me3 as determined by quality metrics and enrichment topology plots. In particular, enrichment near the TSS was more strongly detected for skin than for any of the TOI or the other “adopted” tissues, suggesting that PE reads may better evaluate the broad repressive mark than SE datasets. With only one tissue evaluated as PE, we cannot exclude the possibility that this enrichment pattern may be skin-specific rather than evidence of a better method for detecting H3K27me3. Although enrichment difficulties have been previously recognized for the broad domains like those of H3K27me3 (Landt et al., 2012; Carelli et al., 2017), investigation of specific ChIP methods for broad histone marks appear to be rare. O'Geen et al. (2011) used both short and long sonication periods to account for the different rates of shearing efficiency for compact versus. open chromatin. They found that the larger DNA fragments after sonication were more enriched for broad repressive histone marks while smaller fragments were more likely to contain active chromatin modifications (O'Geen et al., 2011). Their work suggests that shorter sonication times and stringent size selection may bias ChIP samples toward higher enrichment of regions containing narrow marks at the expense of more condensed areas with broad marks, yet current ChIP-Seq standards do not encourage separate protocols for the different mark topologies (Landt et al., 2012; ENCODE Guidelines for Experiments Generating ChIP-seq Data, 2017). Instead, advances in ChIP-Seq methods have focused on analysis and software development to accommodate the different enrichment levels expected from broad and narrow domains assayed with the same protocol (Zhang et al., 2008; Zang et al., 2009). Future investigations involving H3K27me3 and other broad histone modifications may benefit from developing bench protocols, including sequencing parameters, that are specific for broad marks.

To account for insufficient H3K27me3 signal from spleen tissue, IP and sequencing were repeated for both biological replicates. By combining the reads from both sets of data for ECA_UCD_AH2, we were able to obtain sufficient enrichment for peak identification. These data support that combining results from different IPs performed on the same tissue sample can be a useful approach to obtain the enrichment needed for annotation purposes. Study of the best means for combining information from biological and technical replicates for differential enrichment analyses suggests that combining ChIP datasets without accounting for enrichment levels may lead to more false negatives (Bao et al., 2013). Although our data may not have captured all possible peaks, combining data enabled detection of more H3K27me3 peak calls with higher consistency than possible with the first dataset alone. Therefore, the current peak calls can serve as the starting point for spleen-specific annotations, which can be improved upon with characterization of heterochromatin regions from additional equine spleen samples.

The low quality metrics for three of the four marks from ECA_UCD_AH1 sesamoid tissue indicated there was low IP enrichment. To the best of the authors' knowledge, the MC3 and sesamoid data generated here represent the first histone mark peak calls from healthy, whole bone tissue. The overall lower quality metrics for bone tissues support the difficulty of working with these tissues, however, one of the two replicates for sesamoid showed sufficient quality for all four marks, suggesting the issue may be sample specific. To determine if any issues arose during chromatin extraction or IP, further evaluation of H3K4me1, H3K27ac, and H3K27me3 marks in sesamoid tissue from ECA_UCD_AH1 is warranted. Additional data generated from ECA_UCD_AH1 sesamoid tissue will be added to PRJEB42315 when available.

Previous equine annotations were developed based on homology and transcriptomics, leaving much of the genome, especially noncoding regions, uncharacterized (Hestand et al., 2015; Aken et al., 2016; Mansour et al., 2017). While valuable, annotation of regulatory regions based solely on homology with other species is not expected to be sufficient given the evolutionary role of these elements within and among species (Schmidt et al., 2010; McLean et al., 2011; Shibata et al., 2012; Lowdon et al., 2016). With the first publication of the equine FAANG data from eight prioritized tissues (Kingsley et al., 2020) and the four “adopted” tissues presented in this manuscript, researchers can begin to interrogate the role of regulatory regions in equine traits, such as the recent investigation of a novel 16 KB deletion associated with an ocular disorder known as distichiasis (Hisey et al., 2020). Future annotations for the horse will include maps of regulatory states characteristic of healthy tissue, making it a vital resource to compare against disease states. The histone ChIP-Seq data from the horse have already been integrated into a useable annotation resource by a new project known as FAANGMine (FAANGMine, FAANGMine). Similar to FlyMine (Lyne et al., 2007), the project aims to combine the results from all of the genomic assays used by the FAANG consortium into a single resource for easier use. Thanks to these integration effort, additional equine FAANG datasets including the “adopted” tissue peak calls will open up opportunities for variant investigations in previously uncharacterized noncoding regions and expand research opportunities in equine omics.

## Data Availability Statement

Data were submitted to the European Nucleotide Archive following the best practices established by the FAANG Metadata and Data Sharing Committee and the FAANG Data Coordination Centre (Harrison et al., 2018). All of the new data referenced in the article were submitted under project ID PRJEB42315. The following files types were submitted for all high quality data: raw fastq for each mark and input from both replicates (38 files), processed BAM files for each mark and input from both replicates (34 files), bed files with peak calls per replicate including both SICERpy and MACS2 calls for H3K27me3 (32 files), and bed files with combined peak calls including those from both SICERpy and MACS2 for H3K27me3 (16 files). All files and metadata can be accessed from the FAANG Data Portal (https://data.faang.org/home). Previously published FAANG data used in the comparisons are also available from the FAANG data portal under project ID PRJEB35307 (Kingsley et al., 2020).

## Ethics Statement

The animal study was reviewed and approved by University of California, Davis Institutional Animal Care and Use Committee.

## Author Contributions

CF, RB, JP, TK, JM, NH, GL, LO, SB, MM, and EB: conceptualization. NK, RB, CF, and JP: methodology. NK: formal analysis, investigation, data curation, and visualization. CF, RB, and JP: resources. NK and RB: writing—original draft preparation. CF, JP, JM, TK, NH, GL, LO, SB, MM, and EB: writing—review and editing. RB: supervision. CF, RB, and JP: project administration. CF, RB, JP, TK, JM, NH, GL, LO, EB, SB, and MM: funding acquisition. All authors have read and agreed to the published version of the manuscript.

## Conflict of Interest

The authors declare that the research was conducted in the absence of any commercial or financial relationships that could be construed as a potential conflict of interest.
