# Supplementary material for: “Adopt-a-Tissue” Initiative Advances Efforts to Identify Tissue-Specific Histone Marks in the Mare
Source: Front Genet. 2021 Mar 26;12:649959. doi: 10.3389/fgene.2021.649959 (PMC8033197; doi:10.3389/fgene.2021.649959)
Supplement: Supplementary file 1 [file Data_Sheet_1.PDF]

Supplementary Table 1: Metadata for the four "adopted" tissues including specimen details and dissection locations. The identifiers listed for each biological replicate are the ENA Biosample ID for each tissue specimen.

General Term	Specimen Details	ECA_UCD_AH1 Location	ECA_UCD_AH1 Identifier	ECA_UCD_AH2 Location	ECA_UCD_AH2 Identifier
MC3	diaphysis of metacarpal 3 bone	right forelimb	SAMEA104728737	left forelimb	SAMEA104728803
Sesamoid	sesmoid bone	right forelimb	SAMEA104728750	left forelimb	SAMEA104728908
Skin	full thickness skin	dorsal	SAMEA104728773	dorsal	SAMEA104728858
Spleen	spleen	tail of spleen	SAMEA104728847	tail of spleen	SAMEA104728710

Supplementary Table 2: Alignment statistics for all "Adopted" tissue datasets. Values represent the number or percent of reads retained after each major processing step.

Mark	Tissue	Rep	Raw Reads	Aligned Reads	Fraction Aligned	Filtered Reads	Fraction Retained	Duplicates Removed	Fraction Retained	Mapping Quality
H3K4me1	MC3	AH1	54299480	53060859	0.977	44609686	0.822	32624171	0.601	39.3
H3K4me1	MC3	AH2	44609955	43483449	0.975	36581241	0.820	27841274	0.624	39.3
H3K4me3	MC3	AH1	48073400	46951130	0.977	40820791	0.849	5048151	0.105	39.2
H3K4me3	MC3	AH2	51681338	50427310	0.976	43965079	0.851	13702423	0.265	39.2
H3K27ac	MC3	AH1	54107393	52847451	0.977	45540327	0.842	11875218	0.219	39.3
H3K27ac	MC3	AH2	43737105	42737401	0.977	36508428	0.835	2732504	0.062	39.3
H3K27me3	MC3	AH1	119854288	116591477	0.973	90227468	0.753	51165282	0.427	39.3
H3K27me3	MC3	AH2	110859932	107874788	0.973	84810177	0.765	54593706	0.492	39.4
Input	MC3	AH1	84572411	82335647	0.974	65019158	0.769	55694485	0.659	39.2
Input	MC3	AH2	84672593	82497961	0.974	65810982	0.777	56895483	0.672	39.2
H3K4me1	Sesamoid	AH1	45891896	44875523	0.978	38004664	0.828	23620876	0.515	39.2
H3K4me1	Sesamoid	AH2	52938667	51796071	0.978	44032184	0.832	37019848	0.699	39.3
H3K4me3	Sesamoid	AH1	40078088	39146935	0.977	34082271	0.850	12181511	0.304	39.1
H3K4me3	Sesamoid	AH2	46893954	45848365	0.978	39158317	0.835	14945410	0.319	39.2
H3K27ac	Sesamoid	AH1	63131652	61674003	0.977	51954351	0.823	21191826	0.336	39.2
H3K27ac	Sesamoid	AH2	60028953	58736109	0.978	49880778	0.831	16159281	0.269	39.3
H3K27me3	Sesamoid	AH1	57868687	56320918	0.973	44163981	0.763	29691030	0.513	39.3
H3K27me3	Sesamoid	AH2	110579568	108193972	0.978	90068940	0.815	66772818	0.604	39.3
Input	Sesamoid	AH1	76829554	74802343	0.974	59669571	0.777	49465708	0.644	39.2
Input	Sesamoid	AH2	80363657	78426988	0.976	65639004	0.817	54494081	0.678	39.2
H3K4me1	Skin	AH1	101045990	98504165	0.975	91318474	0.904	38495428	0.381	36.0
H3K4me1	Skin	AH2	87209338	85457820	0.980	79459886	0.911	41741916	0.479	36.1
H3K4me3	Skin	AH1	93328152	91165019	0.977	85519746	0.916	43212672	0.463	36.0
H3K4me3	Skin	AH2	108342810	106140174	0.980	99067992	0.914	36666768	0.338	35.9
H3K27ac	Skin	AH1	98203170	96404211	0.982	90251692	0.919	47614788	0.485	36.0
H3K27ac	Skin	AH2	84671932	83069754	0.981	78463594	0.927	47724734	0.564	36.1
H3K27me3	Skin	AH1	235278636	230117904	0.978	199467262	0.848	99953752	0.425	35.8
H3K27me3	Skin	AH2	216721712	211810391	0.977	186085566	0.859	93574734	0.432	35.8
Input	Skin	AH1	237940562	233135467	0.980	198923286	0.836	125342988	0.527	35.8
Input	Skin	AH2	215686900	211396863	0.980	184358841	0.855	111272071	0.516	35.8
H3K4me1	Spleen	AH1	63601827	62745768	0.987	56773348	0.893	36256567	0.570	39.4
H3K4me1	Spleen	AH2	51272309	50434535	0.984	45441490	0.886	33243566	0.648	39.4
H3K4me3	Spleen	AH1	50031817	49263669	0.985	44735618	0.894	27289907	0.545	39.2
H3K4me3	Spleen	AH2	65293465	64232543	0.984	58089426	0.890	32607222	0.499	39.2
H3K27ac	Spleen	AH1	51164015	50539219	0.988	46131632	0.902	29939731	0.585	39.4
H3K27ac	Spleen	AH2	51069779	50403653	0.987	45860424	0.898	31815423	0.623	39.4
H3K27me3	Original Spleen	AH1	120011603	117467034	0.979	98010079	0.817	41090124	0.342	39.4
H3K27me3	Original Spleen	AH2	115571539	113036495	0.978	93613695	0.810	40155514	0.347	39.4
Input	Original Spleen	AH1	46798298	45825840	0.979	38503667	0.823	29042532	0.621	39.4
Input	Original Spleen	AH2	54580462	53467507	0.980	44863485	0.822	33878779	0.621	39.4
H3K27me3	Repeated Spleen	AH1	65517848	62189426	0.949	50991577	0.778	6163159	0.094	39.3
H3K27me3	Repeated Spleen	AH2	83105559	81166884	0.977	66009598	0.794	43415707	0.522	39.3
Input	Repeated Spleen	AH1	96690152	94676056	0.979	78272613	0.810	57791568	0.598	39.3
Input	Repeated Spleen	AH2	77069273	75488501	0.979	62505669	0.811	49284477	0.639	39.3
H3K27me3	Merged Spleen	AH1	185529451	179656466	0.968	149001655	0.803	47153968	0.254	39.4
H3K27me3	Merged Spleen	AH2	198677098	194203397	0.977	159623293	0.803	82873895	0.417	39.4
Input	Merged Spleen	AH1	143488450	140501900	0.979	116776284	0.814	86230894	0.601	39.3
Input	Merged Spleen	AH2	131649735	128956013	0.980	107369154	0.816	82571243	0.627	39.4

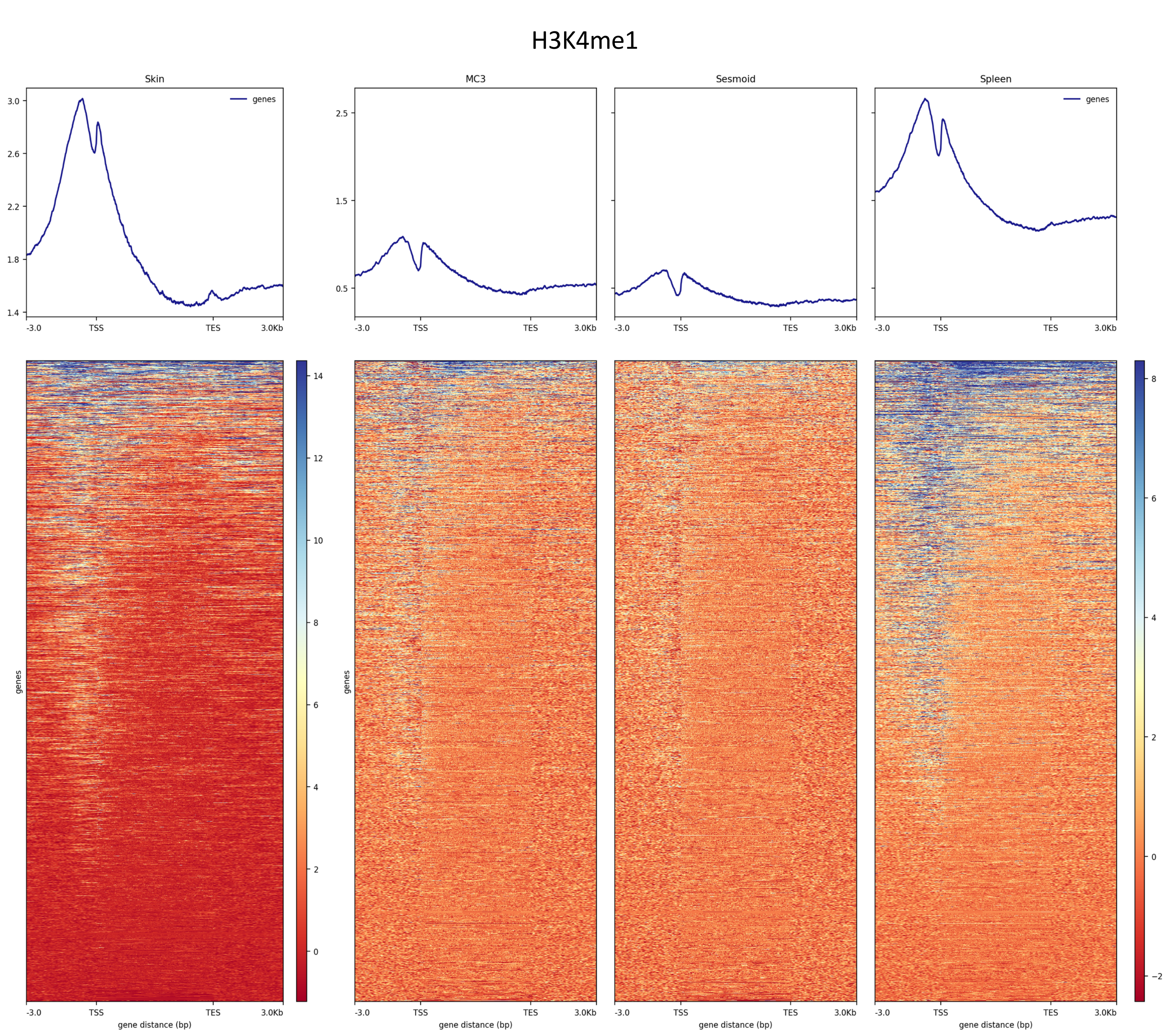

Supplementary Figure 1: H3K4me1 histone mark enrichment across the average gene body. For each panel, topology plots (top) and heat maps (bottom) display the average enrichment for this mark in each of the corresponding tissues across a size-normalized gene distribution based on ENSEMBL annotation (release 95) for EquCab3. Each line in the heatmap represents relative enrichment across a given gene. Presented are the enrichment topologies for skin, MC3, sesamoid, and spleen.

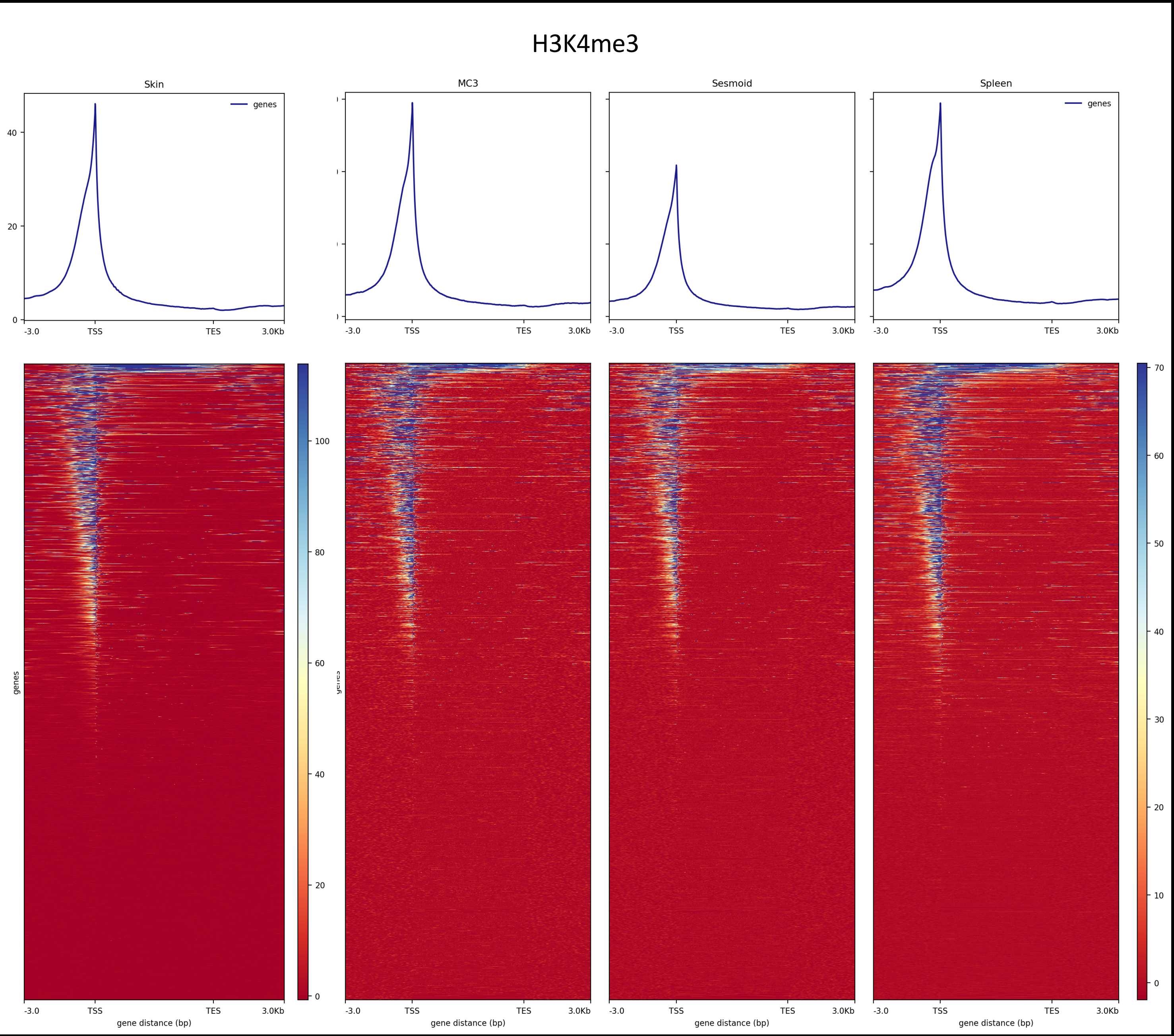

Supplementary Figure 2: H3K4me3 histone mark enrichment across the average gene body. For each panel, topology plots (top) and heat maps (bottom) display the average enrichment for this mark in each of the corresponding tissues across a size-normalized gene distribution based on ENSEMBL annotation (release 95) for EquCab3. Each line in the heatmap represents relative enrichment across a given gene. Presented are the enrichment topologies for skin, MC3, sesamoid, and spleen.

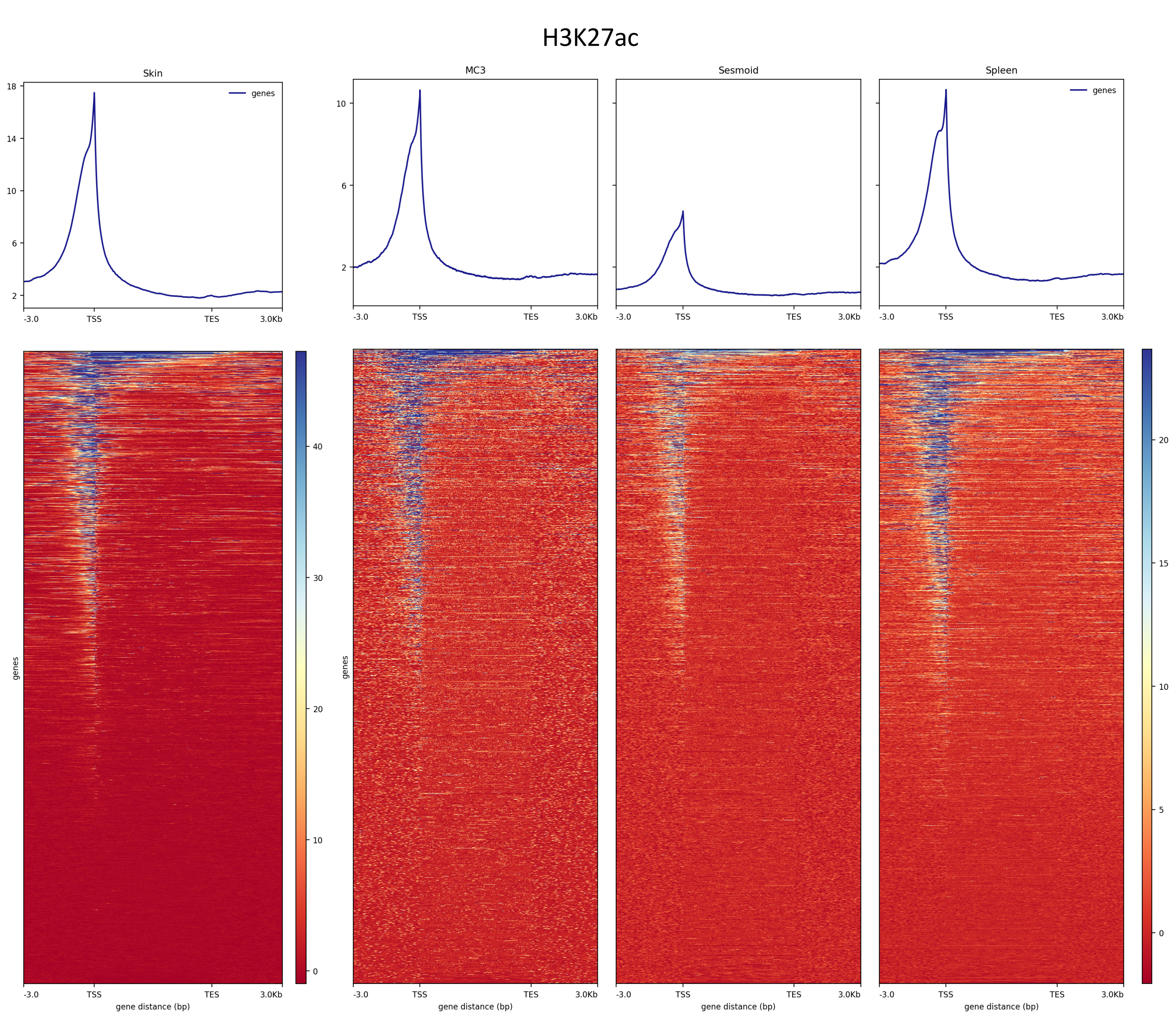

Supplementary Figure 3: H3K27ac histone mark enrichment across the average gene body. For each panel, topology plots (top) and heat maps (bottom) display the average enrichment for this mark in each of the corresponding tissues across a size-normalized gene distribution based on ENSEMBL annotation (release 95) for EquCab3. Each line in the heatmap represents relative enrichment across a given gene. Presented are the enrichment topologies for skin, MC3, sesamoid, and spleen.

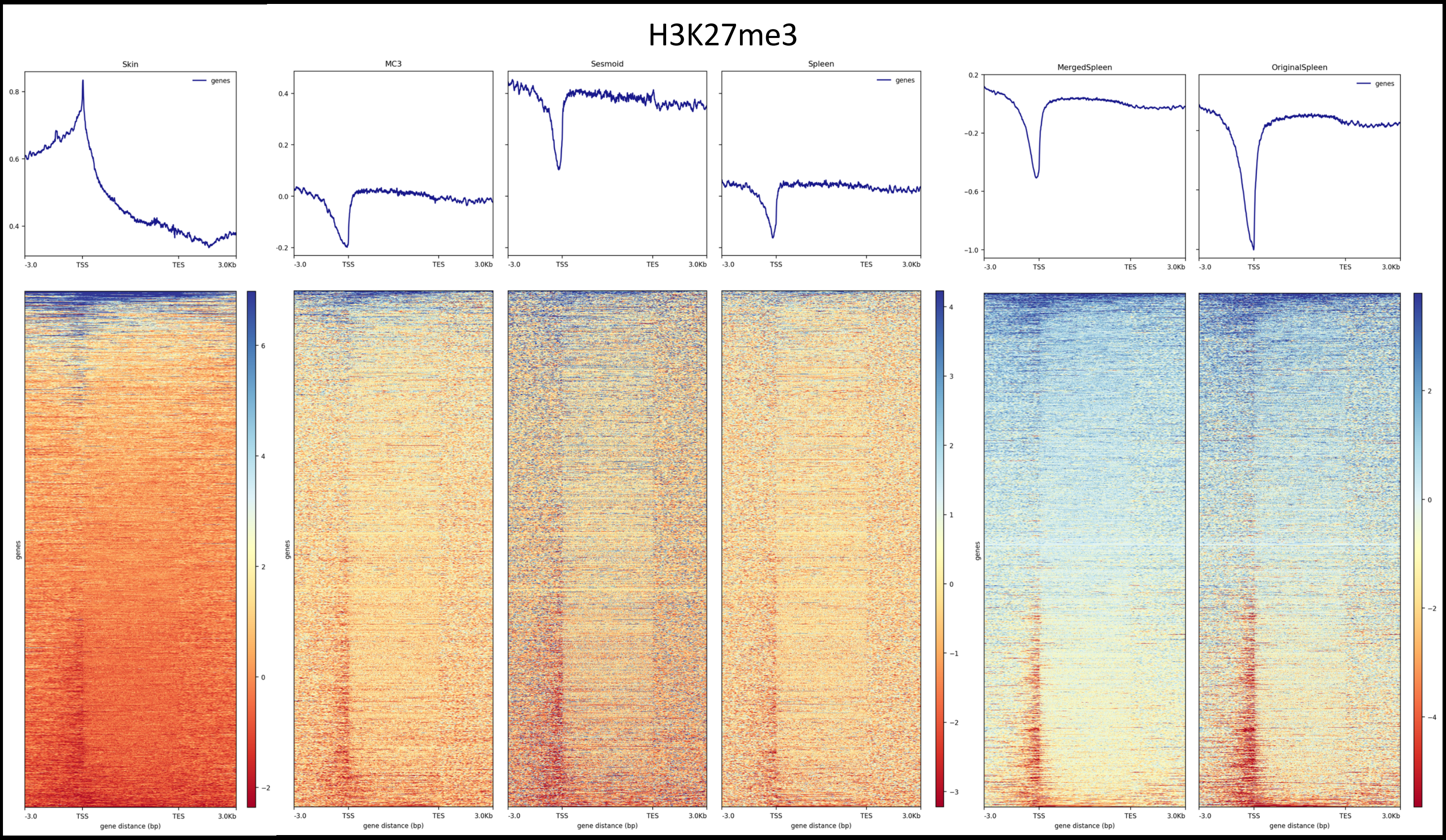

Supplementary Figure 4: H3K27me3 histone mark enrichment across the average gene body. For each panel, topology plots (top) and heat maps (bottom) display the average enrichment for this mark in each of the corresponding tissues across a size-normalized gene distribution based on ENSEMBL annotation (release 95) for EquCab3. Each line in the heatmap represents relative enrichment across a given gene. Presented are the enrichment topologies for skin, MC3, sesamoid, spleen. Given that this mark performed poorly for spleen tissue initially, the experiments were repeated, and the data were analyzed multiple ways. The plot noted as "Spleen" represents the AH1 data from initial IP and AH2 merged data for both the initial and repeat IP experiments. "MergedSpleen" represents merged data (initial IP and repeat IP experiments) from both replicates. The "OriginalSpleen" plot represents data only from the initial IP and sequencing experiment for both biological replicates.

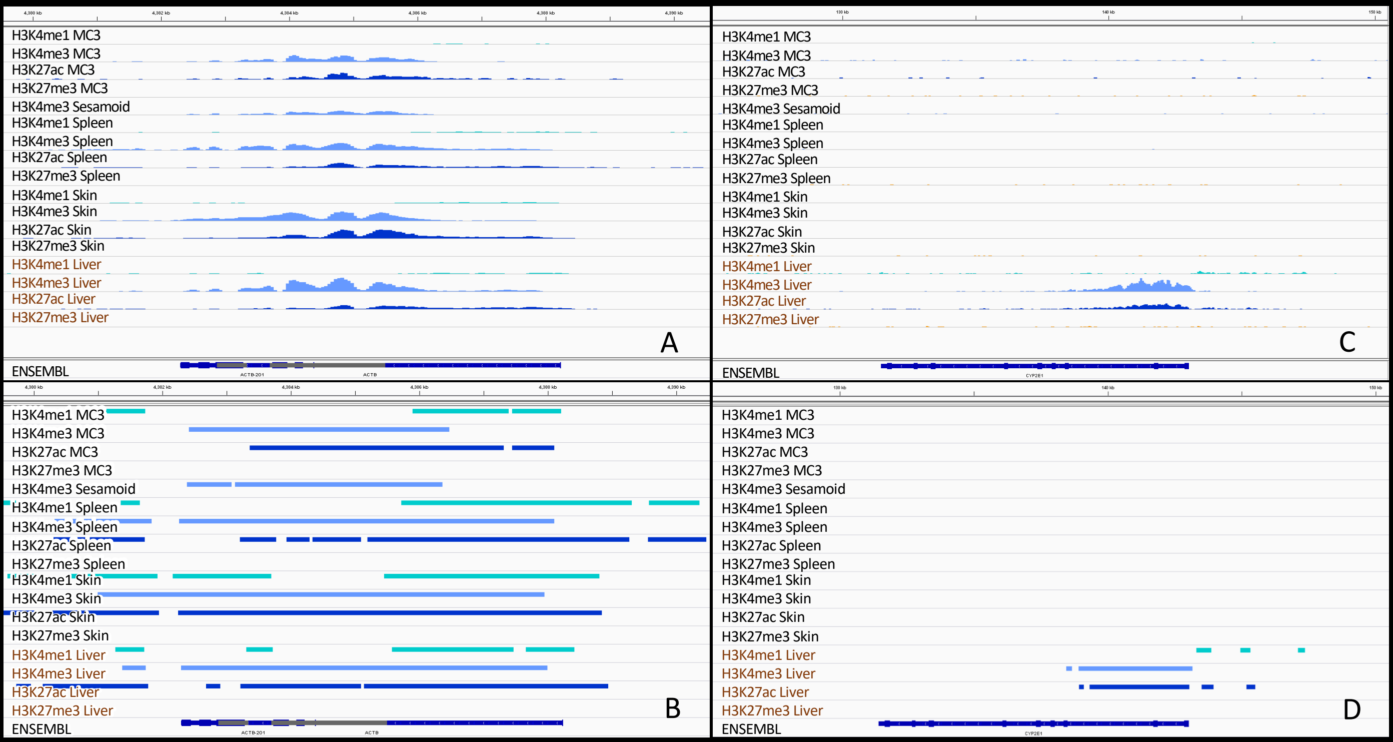

Supplementary Figure 5: Histone mark enrichment and peak calls from four "adopted" tissues and previously characterized liver data for one house-keeping gene and one liver-specific gene. Evaluation of high-quality data was performed using Integrated Genome Viewer. ChIP enrichment tracks were generated as bigWig files in which the input alignments were subtracted from the IP read distributions prior to combining both biological replicates for each mark. All peak calls were visualized from BED files. H3K27me3 peaks were called with SICER, and all other marks were called with MACS2. (A) ChIP enrichment detected in region surrounding house-keeping gene, *ACTB.* (B) Replicate-combined peak calls for the same region from panel A. (C) Enrichment of ChIP and (D) replicate-combined peaks detected for tissue-specific gene previously identified for liver tissue, *CYP2E1*.
